# The post-scarcity world and the post-pandemic office

**DOI:** 10.3389/frma.2022.976798

**Published:** 2022-08-16

**Authors:** Salil K. Mehra

**Affiliations:** Beasley School of Law, Temple University, Philadelphia, PA, United States

**Keywords:** scarcity, regulation, abundance, economics, competition, government, taxation

## Abstract

We are not yet in the post-scarcity world that John Maynard Keynes famously envisioned, and vaccines have only recently allowed us to hope that a post-COVID-19 future may arrive soon. However, it is not too early to consider the impact of both on the traditional office, and on attempts to bring it back for reasons that may be socially harmful. One lesson of the pandemic is that many workers can be as—or even more—productive working from home, thanks chiefly to software such as Zoom, Microsoft Teams, and Slack, among others, which enable better collaboration across distances than was previously possible. At the turn of the century, we moved toward an economy in which important products were increasingly characterized by low marginal costs of production, such as pharmaceuticals and software. Over the past decade, we have seen fixed costs reduced in some situations—consider how Uber greatly eliminates the need for a central taxi dispatcher, and makes use of idle capital invested in personal vehicles. The traditional office represents a massive fixed cost for many industries; tech-driven work-from-home greatly reduces the need for this fixed cost. While software, Internet connectivity and the cloud are not free, preliminary estimates suggest that replacing traditional offices with work-from-home greatly lowers costs, creates economic efficiencies and, relatedly, reduces environmental harm. That said, the story of work-from-home is not one of unbridled optimism. Real estate firms and local governments are already trying to use law as a tool to return workers to the pre-pandemic traditional office. Various levels of government seek to return workers to physical offices, often motivated by declines in tax receipts. Attempts to bolster a return to the traditional office may raise fixed costs for firms and generate substantial avoidable environmental damage. This Chapter recommends competition advocacy to counterbalance state and local attempts to prevent the efficient disruption of the traditional office's fixed costs. Work-from-home represents an important step toward the post-scarcity world; but without a focus on what amounts to state-and-local protectionism in this sphere, we could wind up taking another step backwards.

## Introduction

An Internet-famous meme centers on a picture of the town of Breezewood, Pennsylvania, depicting a landscape of fast-food and service station logos to critique what, it is alleged, capitalism has done to the American landscape.^1^ The photo depicts a brief stretch of local road that many of those traveling between Interstate 70 (“I-70”) from Washington, Baltimore and points southeast must cross to switch to the Pennsylvania Turnpike westbound toward the Midwest. But while a picture may be worth a thousand words, this picture and those words may be deceiving. The gas station and fast food dominated landscape pictured is not the work of unfettered free-market capitalism. Instead, it is capitalism mixed inseparably with the unintended consequences of law, at the federal, state and local levels.^2^ At the federal level, the National Interstate and Defense Highway Act of 1956 prohibited the use of federal funding to directly connect the then-new Interstates such as I-70 with pre-existing toll roads such as the Pennsylvania Turnpike, which opened in 1940.^3^ Accordingly, the Commonwealth of Pennsylvania used the provided funds to create an indirect connection whereby, for few miles, drivers from toll-free I-70 would ride on a local road before accessing the Turnpike. Over time, this stretch of local road became a chronic traffic-jammed bottleneck that attracted profit-seeking businesspeople seeking to lure slowed motorists to pull over and fill their gas tanks and stomachs. While the federal law preventing a direct connection between these expressways has since been repealed, Pennsylvania's legal process for considering new highway improvements requires that such changes be first proposed by local governments.^4^ And tax revenue, employment and voting considerations being what they are, no local elected official is going to propose a bypass that would bankrupt a significant percentage of their town's employers.

As we recover from the COVID-19 pandemic, a similar dynamic could take place involving a different set of places of employment: office buildings. This shift has reduced the consumption of time and fuel for commuting, the cost of rent for corporate shareholders, and the damage of carbon emissions for the planet. The pandemic has shown that a substantial number of office employees could work productively from home. But federal, state and local laws produced the office landscape, and the force of incumbent arrangements may seek to return office workers to those sites.

Like Breezewood, these forces may make the pre-pandemic office harder to escape than it should be. That would be unfortunate. One of the few silver linings of the COVID-19 pandemic was the way it made Americans reconsider existing arrangements, especially the weaknesses of our healthcare system. Similarly, COVID-19 has forced firms and employees to reconsider whether they can avoid the expense of their pre-pandemic office space by continuing to work from home. Doing so would significantly lower the cost of production for many firms—and by doing so reduce economic scarcity.

We are not yet in the post-scarcity world that John Maynard Keynes famously envisioned, and vaccines have only recently allowed us to hope that a post-COVID-19 future may arrive soon. However, it is not too early to consider the impact of both on the traditional office, and on attempts to bring it back for reasons that may be socially harmful. One lesson of the pandemic is that many workers can be as—or even more—productive working from home, thanks chiefly to software such as Zoom, Microsoft Teams, and Slack, among others, which enable better collaboration across distances than was previously possible. At the turn of the twenty-first century, we moved toward an economy in which important products were increasingly characterized by low marginal costs of production, such as pharmaceuticals and software. Over the past decade, we have seen *fixed* costs reduced in some situations—consider how Uber greatly eliminates the need for a central taxi dispatcher, and makes use of idle capital invested in personal vehicles. The traditional office represents a massive fixed cost for many industries; tech-driven work-from-home greatly reduces the need for this fixed cost. While software, Internet connectivity and the cloud are not free, preliminary estimates suggest that replacing traditional offices with work-from-home greatly lowers costs, creates economic efficiencies and, relatedly, reduces environmental harm.

That said, the story of work-from-home is not one of unbridled optimism. Real estate firms and local governments are already trying to use law as a tool to return workers to the pre-pandemic traditional office. For example, in *New Hampshire* v. *Massachusetts*, Massachusetts sought to continue levying income tax residents of New Hampshire working from their homes in the latter state, if they worked in physical offices in Massachusetts prior to the pandemic's start in March 2020; a Massachusetts victory would have eliminated some of the private economic savings due to work-from-home, reducing the incentive to continue it.^5^ Various city governments have lobbied both the federal government and private firms to return workers to physical offices, often with the goal of gathering local income and sales taxes from those workers, not to mention bolstering property tax receipts. While there is nothing intrinsically wrong with state and local governments seeking tax revenue, the attempts to bolster a return to the traditional office may raise fixed costs for firms and generate substantial avoidable environmental damage.

Additionally, and more abstractly, law provides a variety of hidden subsidies to the traditional office. By failing to consider these as costs, we risk undervaluing the gains from work-from-home as a disruptive innovation. That said, this transition creates winners *and* losers; steps should be taken to reduce harms that would increase inequality.

This Chapter suggests a program of antitrust law and competition advocacy aimed at fostering opposition to state and local attempts to prevent the efficient disruption of the traditional office's fixed costs. Removing the office's fixed costs would not only be efficient for employers; it would also produce benefits for employees and the environment. Work-from-home represents an important step toward the post-scarcity world; but without a focus on what amounts to state-and-local protectionism in this sphere, we could wind up taking another step backwards.

## What is an office? Is it a “place where dreams come true”?

“Nobody should have to go to work thinking, ‘Oh, this is the place that I might die today.' That's what a hospital is for. An office is for not dying. An office is a place to live life to the fullest, to the max, to… An office is a place where dreams come true.”

–Michael Scott (played by Steve Carell), The Office^6^

Until very recently, the office has been a central setting for American life. Over 130 years ago in Chicago, the first building to be called a ‘skyscraper' was completed as offices for the Home Insurance Company. An office building with over a 90 percent occupancy rate before the Great Depression, it was demolished in 1931.^7^ By contrast, America's purported first office park, built in the Birmingham, Alabama suburb of Mountain Brook in the 1950s, still exists.^8^

But the temporary closure of many offices due to COVID-19, while the economic life of the country continued, raises the question: Do we still need offices? And even if some of us do, does America still need as many as we have had?^9^ As is well-known, the pandemic spurred a huge spike in working-from-home. Some observers have concluded that some of this shift will be permanent.^10^ An American economy driven by the service sector is centered on offices by choice; one might conclude that whether we still need offices and office buildings should be left to the private decisions of businesses and their workers.^11^ Moreover, the office looms large in American life not only in a physical dimension, but also, some claim, in historical and sociological ones.^12^

However, America is also office-centric because law makes it so, in ways that have been up until now unexamined. States and cities have built their revenue models in part on the assumption that office workers would fund government activity, whether those workers were residents or not.^13^ Federal tax law has been bent to accommodate and promote the office.^14^ These rules are embedded across an array of legal fields, constructing a kind of “office centricity.” Commentators have raised similar arguments about law's hidden subsidies for cars^15^ and sports,^16^ which like office buildings help generate economic activity. However, offices differ from cars and sports in an important respect: most reasonable people do not find offices liberating, entertaining or fun.^17^ While this may seem like a glib observation, it is an important one—fun is a form of utility, and if offices, unlike cars or sports, do not generate much or any nonpecuniary utility, then their only utility is the economic gain that they generate for firms and their employees.^18^

But if the economic activity associated with offices can be accomplished without them, do they need to exist? And is this a question that should involve anyone other than employers and employees? The COVID-19 pandemic has, by revealing the effectiveness of work-from-home arrangements, rendered these questions more than theoretical. And indeed, employers have taken note. Morgan Stanley's CEO has forecast a future with “much less real estate.”^19^ Barclays' CEO has asked whether “the notion of putting 7,000 people in the building may be a thing of the past.”^20^ And Nationwide Insurance, with 32,000 employees, plans to shutter most of its offices.^21^

While surprising to some, in fact, technologists had forecast a shift to what was then called “telework” for half a century. British futurologist James Martin had envisioned in 1970 that “[t]he time will come when the computer terminal is a natural adjunct to daily living,” and as a result “some companies may have almost no offices.”^22^ Similarly, in his 1980 best-seller *The Third Wave*, Alvin Toffler predicted the development of the “electronic cottage,” with computers and telecommunications driving a shift to “the home as the center of society.”^23^ In fact, such a shift would be a homecoming of sorts, as office work was once the province of a rarified few gentlemen in home offices in their mansions and estates.^24^

Seen in this context, the high-rise offices and office-park cubicle farms of the twentieth century are a relatively short-term blip in the history of work. To some extent, its durability is a matter of both aesthetics and law. With regard to aesthetics, the mid-twentieth century German movement of *Bürolandschaft* (“office landscaping”), captivated Anglo-American observers, describing a then-novel arrangement that is familiar to us now:

The receptionist greets us from her desk and while we wait, we have time to look around. The windows seem a long way away… but sunlight can still be seen in the trees and on the roofs of the factories outside. Desks, furniture and equipment are disposed, apparently at random, amid portable acoustic screens and tub plants… Because of the screens and the random disposition of desks, we are not a focus of attention as we make our way down the wide circulation path to the desk of the man we have come to see. That he is a man of some importance is made clear by the fact that he enjoys more space and better equipment than his staff…^25^

A few years later, the Port Authority of New York and New Jersey adopted *Bürolandschaft* as it planned the floors of its then-new, and ill-fated, World Trade Center in lower Manhatttan.

However, high-rise offices and low-rise office parks were not only a product of architectural trends. In fact, law promoted their widespread adoption. The U.S. Revenue Act of 1962 encouraged office landscaping, especially cubicles—a permanent building wall was assigned a life of 39-1/2 years over which its costs could be depreciated, but moveable panels could be depreciated over just seven years, much as typewriters and telephone could.^26^ As a result, tax law encouraged cubicle farms over walls with permanent offices.

Moreover, law's accommodation and promotion of the office goes beyond favorable treatment for cubicles. At the local level, some cities, including New York, Philadelphia and Pittsburgh, started to tax non-resident office workers who commute into the city—creating a strong incentive for these local governments to promote office construction. State governments compete with economic incentives to attract corporate headquarters. And the federal government subsidizes offices in various ways—both directly, in the form of highway and mass transit, and indirectly, by administering a legal regime to deal with conflict that emerges from the office context, including labor, employment discrimination and sexual harassment regimes.^27^ But the question remains: Does it have to be this way?

## The office and the post-scarcity world

“We don't have a lot of time on this Earth! We weren't meant to spend it this way! Human beings were not meant to sit in little cubicles staring at computer screens all day… ”

–from the film *Office Space* (1999)^28^

Most of human existence has been characterized by scarcity—of, among other things, food, water, shelter and time. However, the post-industrial developed world increasingly can meet people's basic needs fairly easily, even if its governments sometimes choose not to. From an economic perspective, a post-scarcity world is one in which goods and services can be produced at costs that approach zero, and where producers accordingly set prices approaching zero. Two assumptions are implicit. First, producers do not possess market power that enables them to earn economic rents.^29^ Additionally, market failures, such as large transaction or switching costs, do not prevent prices from driving toward marginal cost.^30^ Both of these assumptions do not necessarily happen automatically, but are instead propositions that can be realized through the operation of competition law.^31^

**Figure 1:**
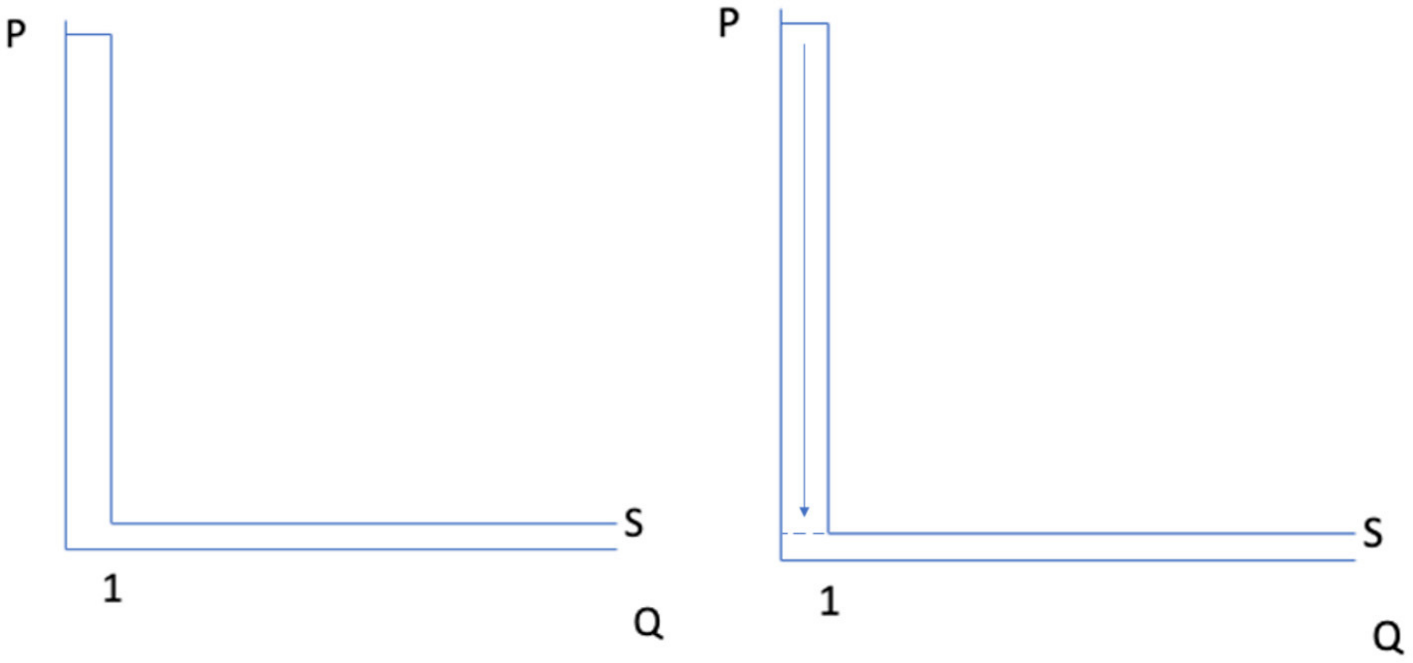
Incremental and fixed costs falling toward zero.

What would it take for us to achieve a post-scarcity world? Costs of production—both fixed and variable—would need to approach zero. Figure 1 illustrates this possibility. On the left side is a diagram of a supply curve for a product for which is extremely expensive to produce the first unit of consumption, but for which the marginal cost of additional units approaches zero. A supply curve like that on the left side of Figure 1 characterizes many situations, including bridges, amusement parks, and perhaps most prominently in the law review literature, intellectual property. Consider as an example, books. The second, third and following copies traditionally cost little to produce. But the first copy required significant effort from the author, as well as, for example, a publisher's investment in a printing press—though the latter might be reused for subsequent books.^32^ This dynamic has traditionally been used to ground copyright law—the price for subsequent copies cannot be set to marginal cost without some way of recouping the large cost of the initial copy, or the work will not be created.^33^

Digitalization in the form of, for example, e-books reduces costs, and as the costs of more and more products fall to zero, we enter a post-scarcity world. Much of the focus on digitalization has pointed to the effects of reduced *variable* costs involving the second, third and following copies—e-books eliminate costs such as paper, binding, shipping and retail shelving for these succeeding copies.^34^ Indeed, the low costs at which additional copies can be made has created a technological challenge for conventional intellectual property law.

However, technological advances such as digitalization also can reduce *fixed* costs, as depicted in the supply curve on the right side of Figure 1. In this example, not only the costs of the succeeding units, but also the first unit of the product start to approach zero, as shown by the arrow pointing down from the original cost of the first unit to the dotted line representing its new, approaching-zero cost. For example, the printing press cost included in the first copy of a book disappears in the context of an e-book. While other costs, such as the author's work, may endure, they too might be reduced if AI succeeds at producing valuable creative work, depending on the relative cost of authors vs. AI.^35^

The potential for first-unit, fixed costs to drive lower, or even toward zero, is not limited to intellectual property. In various fields, technological change is driving down initial fixed costs of production. With Internet connectivity, aspiring journalists no longer need a printing press, and musicians have little to no need for a record press. Before 3D printing, producing a working firearm required at minimum the tools of a gunsmith, if not a factory, depending on the type; now they can be produced with a 3D printer.^36^ Finally, increased interconnectivity and computing power can replace dedicated communications infrastructure. Consider Uber: adding software to pre-existing, general-use, Internet-linked smartphones in the hands of drivers who owned private cars obviated the need for a high-fixed-cost taxi dispatch and radio system, as well as a taxi fleet.

By forcing work-from-home, the COVID-19 pandemic has revealed that something like the changes outlined above may be possible for offices. Many office workers had sufficient space in their existing homes to set up a workspace. High-bandwidth Internet plus easy-to-use software such as Zoom and Microsoft Teams enabled these workers to conduct meetings with their counterparts that, while not physically in-person, were face-to-face, in a manner of speaking.

The work-from-home trend has taken advantage of technologically reduced costs. It has also in turn reduced other costs. Commuting time and expenses has been saved. And those savings have benefitted the environment since they involve reduced carbon emissions and other pollution. Finally, the ability to work from home potentially allows employers and employees to save on office rent, other overhead and taxes. But those incumbents who benefitted from the pre-pandemic status quo have started to resist making the transition to work-from-home permanent.^37^ For landlords, some public officials and others, the social savings are outweighed by their private and local losses; conspicuously, these opponents of work-from-home include government officials from localities that house significant concentrations of pre-pandemic office space.

## Can you work in pajamas at home all day?

“You can't stay at home in your pajamas all day.”

– New York City Mayor Eric Adams^38^

Understandably, New York City's mayor opposes work-from-home. As of 2021, Manhattan by itself accounts for 11% of all U.S. office space.^39^ Additionally, Brooklyn and Queens combined rival Silicon Valley in terms of total office space.^40^ Due to higher property tax assessment rates, Midtown and Downtown Manhattan alone supply more than a quarter of the city's property tax revenue.^41^ Moreover, offices and their workers also produce difficult to measure indirect revenue, including sales taxes from office workers' spending and personal income tax from office workers who choose to live in a city—such as New York—rather than outlying areas to enjoy shorter commutes.^42^ While local officials' calls for a return to the office certainly involve a desire for a return of revenue, they also reflect concerns about the impact of work-from-home on those residents who used to serve office workers, such as restaurant staff, dry cleaners, and the like. Both sets of concerns deserve attention, but not necessarily the same level of sympathy.

### Return to the office: The revenue story

The reasons politicians call for a return to the office are likely the reasons they take many other positions: votes and cash.^43^ First, some cities pre-COVID hosted daytime working populations larger than their resident working-age population.^44^ In such a situation, a city's businesses may become dependent on the commuter population, and those resident businesses may generate local tax revenue and employ a city's voters.^45^ More directly, states and some cities tax non-resident workers who commute into their geographic territory; the transition to work-from-home may remove these workers from the tax base accessible to such state and city politicians.^46^

The desire to tax former commuters now working from homes outside the jurisdiction of their former offices drove the *New Hampshire* v. *Massachusetts* case. Much of New Hampshire “is a bedroom of Massachusetts.”^47^ Pre-COVID, almost triple the number of New Hampshire residents commuted into Massachusetts than made the opposite commute, meaning that, unless the relative salaries were quite different, for each state to be left taxing only its residents working from their homes would be a loss to Massachusetts and a potential gain to New Hampshire—were the latter, like the former, to tax earned income.^48^ As a result, Massachusetts has tried to continue levying income tax on residents of New Hampshire working from their homes. Embodied in an April 2020 emergency ruling by its Department of Revenue, Massachusetts took the unprecented position that the fact that these New Hampshirites had previously commuted to physical offices in Massachusetts prior to the pandemic's start in March 2020 provided sufficient nexus to bring them within Massachusetts' taxing authority.^49^

While the Supreme Court denied New Hampshire's attempt to challenge the constitutionality of Massachusetts' nexus argument on a collective, statewide basis, the argument in the case continues to be litigated on an individual basis by the affected taxpayers. Moreover, Massachusetts discontinued the emergency rule as of September 13, 2021, meaning that it only applies to income during the first 18 months of the pandemic, and going forward, Massachusetts will only tax work done within the state—the prevailing rule nationwide.^50^

That said, the issue at the heart of *New Hampshire* v. *Massachusetts* remains an important one. Several states, including New York, Delaware, and Pennsylvania, tax work done for in-state companies by out-of-state residents.^51^ Whether that is constitutional remains unresolved. And so long as that is so, states and cities will have an incentive to reach—and perhaps overreach—even when doing so is at odds with attempts to lower costs to workers, firms, shareholders, society, and the environment.

### Return to the office: Workers—winners and losers

Like most transformations, the shift to work-from-home creates winners and losers. The gains overall to the winners almost certainly outweigh the costs to the losers. Despite that, a strong distributive equity case argues for addressing those costs that would worsen inequality.

Reduced commuting drives (pardon the pun) some of the largest gains to society from work-from-home. But officials have fallen into the trap of ignoring these costs. For example, in his 2022 State of the Union Address, President Joseph Biden said it was “time for Americans to get back to work and fill our great downtowns again,” and called for a “return to the office.”^52^ This was despite oil prices having moved sharply higher due to Russia's invasion of Ukraine, and the ensuing sanctions imposed by the international community. And a “return to the office” would suggest even higher prices for oil and gasoline; just prior to the pandemic, more than three-quarters of American commuters drove their cars to work alone.^53^ Critically, gasoline is literally a textbook example of an inelastic good—one which consumers are relatively unlikely to cut back on even as prices rise. ^54^ Such inelasticity also implies that gasoline prices will rise substantially if, all other things being equal, consumers increase their consumption, as they may do if forced to return to the office.^55^ Moreover, gasoline is only one cost for most American commuters. The commute also involves environmental and social costs that include carbon emissions and the physical and legal infrastructure that subsidizes commuting.^56^ These are all costs that work-from-home can reduce.

Moreover, the traditional office relies on the legal system for support, and this support has costs. Aside from the tax incentives discussed previously, the traditional office involves a system of hierarchy that can foster abuse. Late twentieth century scholarship at the dawn of sexual harassment litigation grappled with its nexus with the “workplace.”^57^ It may be that the workplace should not be defined exclusively as a geographic real-world site.^58^ While harassment and discrimination will not disappear in lockstep with the traditional office, some egregious conduct that law polices will not be possible without physical proximity.^59^ Law's role in patrolling the boundary between permissible and impermissible uses of hierarchy in the traditional office has economic and social costs; courts are taxpayer subsidized, and the time and money used involves the costs of foregone opportunities for the legal system to address other problems.

However, there are losses to some from the transition to work-from-home. Some of those who shift to work-from-home may suffer from that change. During the first year of the COVID-19 pandemic, working mothers were disproportionately made to combine both work-from-home and remote school for their children, a situation that reduced their workforce participation and imposed significant but as-yet-uncertain costs on them.^60^ Going forward, it bears paying attention to whether work-from-home generates significant inequality on its own even after other COVID-19-related effects, such as remote schooling, are no longer present.

Additionally, the transition to work-from-home will cost those workers who served the traditional office, for example by cooking or cleaning for office-bound employees, or in other indirect ways. Addressing their economic distress does not necessarily require a broad-based bailout of landlords in and governments of relatively rich coastal cities such as New York and San Francisco. Indeed, doing so would likely exacerbate existing economic inequality at a national level. But a smoother transition to a post-scarcity work in the office context may require policies to aid injured workers. Other structural adjustments, such as exposure to foreign imports from lower-wage countries or increased use of robotics, have been accompanied by steps to help workers transition; a similar approach focused on preventing increased inequality seems well-advised now.

## Thinking outside the cube: Preventing anti-disruption

[*Bill sets up a cubicle around his desk]*

Dave: “Have you thought about how this will make your co-workers feel?”

Bill: “Actually, one of the great things about the cubicle is not having to think about my co-workers at all.”

–*Newsradio*, episode 2.5^61^

State and local officials seeking to return private sector workers to offices, or retain their tax revenue as if they did return, echo the political forces that keep Breezewood a traffic bottleneck between the Southeast and the Midwest. Breezewood's politicians understandably focus on local revenue and employment. But in doing so, they ignore costs such as drivers' lost time and idling cars' carbon emissions. And those costs may greatly exceed the local benefits of preventing a smooth junction for travelers from the East to the Midwest.

Similarly, state and local politicians from areas with pre-pandemic concentrations of offices have focused on retaining commuter generated revenue, especially by seeking to return those commuters to the office. As with Breezewood, while the costs of forcing workers back to the office may exceed the local benefits, the political calculus may diverge from overall social welfare. That said, because the COVID-19 pandemic has made firms, shareholders and employees recognize that there are gains to be had by shifting permanently to work from home and hybrid work, there may be a more organized pushback to a forced office return than to keeping Breezewood's status quo.

In a sense, the COVID-19 pandemic has “disrupted” the traditional office, and some state and local politicians are responding with moves aimed at “anti-disruption,” such as trying to engineer a physical return, and, as in Massachusetts' case, seeking to tax workers outside their borders.^62^ Government has intervened to try to block disruptive innovation such as Internet-powered work-from-home before; consider the case of Uber and other ridesharing services.^63^ As work-from-home does with employers and workers, when ridesharing appeared on the scene it facilitated transactions that benefitted buyers and sellers—but government sometimes sought to alter or stop those transactions. Seattle's City Council passed an ordinance authorizing collective bargaining by drivers under an “exclusive driver representative” seeking to raise drivers' wages and thus Uber's costs; after the 9th Circuit ruled that the ordinance might be preempted by the Sherman Act, the city “tweaked” the law and settled with Uber and other private plaintiffs.^64^ In a similar vein, Massachusetts' actions in *New Hampshire* v. *Massachusetts* reduced the economic gains to employers and workers from work-from-home by eliminating tax savings. The Philadelphia Parking Authority (“PPA”), which also regulates and collects revenue from traditional yellow cabs, hired lobbyists to influence the state legislature not to legalize Uber, while simultaneously organizing taxi companies to run undercover stings on Uber drivers and report them to the police.^65^ The PPA's resort to use of armed police forces resembles the heavy hand of government coercing private employees back to their firms' offices.

Attempts by state and local government to thwart or reverse work-from-home arrangements may take different forms than anti-disruptive activity against ridesharing. As the example of Breezewood suggests, significant local benefits can drive politicians to force others to incur even greater global costs. But such anti-disruption can be resisted, if not prevented. First, antitrust law can be activated to preempt or restrict state or local government action that thwarts market ordering; the Supreme Court has signaled increased skepticism of such regulatory action.^66^ Second, when possible, the Dormant Commerce Clause should be used to limit attempts by state or local government to expand their authority in ways that reduce competition between jurisdictions over taxes, regulation or other matters. Finally, competition advocacy requires that agencies, academics and thought leaders engage in ways that educate the public on the harm that can result from preventing private actors from reordering their affairs in accord with their own costs and benefits.

## Conclusion

The discussion here is by necessity tentative. The COVID-19 pandemic is not yet over, so the post-pandemic workplace is still largely a forecast. However, the political forces that would try to return workers and offices to the status quo pre-pandemic—notwithstanding the costs in time, money, and environmental damage—have started to show themselves. Law already has tools aimed at cabining politicians' attempts to gain locally at global expense; legal actors must ready and willing to use them.

## Data availability statement

The original contributions presented in the study are included in the article/supplementary material, further inquiries can be directed to the corresponding author/s.

## Author contributions

The author confirms being the sole contributor of this work and has approved it for publication.

## Funding

I have received summer research funding from Temple University, James E. Beasley School of Law.

## Conflict of interest

The author declares that the research was conducted in the absence of any commercial or financial relationships that could be construed as a potential conflict of interest.

## Publisher's note

All claims expressed in this article are solely those of the authors and do not necessarily represent those of their affiliated organizations, or those of the publisher, the editors and the reviewers. Any product that may be evaluated in this article, or claim that may be made by its manufacturer, is not guaranteed or endorsed by the publisher.

## Footnotes

^1^Hurley (2019) (showing picture by Edward Burtynsky, courtesy Nicholas Metivier Gallery, Toronto).

^2^*Id.;* Savage (2017).

^3^Federal Aid Highway Act of 1956 (better known as the “National Interstate and Defense Highways Act”), Public Law 84-627, June 29, 1956.

^4^Savage, *supra* n. 4.

^5^See *infra* nn. 44–52 and surrounding text.

^6^The Office (US TV Series), Season 5, Episode 13, “Stress Relief” (note that this quote is said by a character who is deluded to a cringeworthy degree).

^7^Kamin (2019).

^8^Marshall (2016) and The Historical Marker Database (2016) (listing and describing historical marker for “America's First Office Park”).

^9^But Patail (2019) (asking this question even before COVID-19's U.S. impact).

^10^Barrero et al. (2021) (describing economic incentives favoring continued work-from-home post-pandemic).

^11^Out of all commercial real estate in the U.S.—a category that includes hotels, malls, big box stores such as WalMart, restaurants, apartments, factories, warehouses, as well as nonprofits like schools and hospitals—almost a third by square footage consists of office buildings (Amadeo, 2022).

^12^*See, e.g.*, Saval (2014) (describing the mid-twentieth century's office's impact on shaping the American white collar worker's norms).

^13^*See infra* nn. 44–47 and surrounding text.

^14^*See infra* nn. 26–27 and 58–61 and surrounding text.

^15^*See* Shill (2020) (arguing that a “structure [of] ‘automobile supremacy'… constructed by diverse bodies of law” “shift[s] costs” and “legitimate a state of choice deprivation and inequity”).

^16^*See* Haddock et al. (2013) (describing sports stadia as publicly subsidized due to a competition problem at the intersection of political and market structures).

^17^*See supra* n. 2.

^18^There are those who have in the past lauded the interpersonal relationships that can flourish in the office environment; at least some of those relationships would run afoul of contemporary sexual harassment laws. *See, e.g.*, Brown (1962) (advocating strategic use of office sexual relationships).

^19^Haigh (2020).

^20^*Id*.

^21^Id. at 5.

^22^Id. at 10 [quoting Martin and Norman (1970)].

^23^*Id*.

^24^*Id*. at 16–17 (describing such a home office in an eighteenth century historic home of a merchant and financier of the whaling trade in Marblehead, Massachusetts).

^25^*Id*. at 61 [quoting *Architectural Review* (1964)].

^26^Petersen and Warzel (2021).

^27^U.S. Equal Employment Opportunity Commission (2022) (noting that “[h]arassment is a form of employment discrimination that violates Title VII of the Civil Rights Act of 1964,” among other laws).

^28^Judge, M. (Director) (1999), *Office Space* (twentieth Century Fox).

^29^Mehra (2016).

^30^*Id*.

^31^*Id*.

^32^Originally, however, typesetters produced pages by setting blocks by hand. While the blocks could be reused, the first-copy cost per work was quite high—though not as high as before moveable type and printing presses (Marech, 2014) (describing operation of Ben Franklin's press).

^33^Gordon (1992).

^34^Lemley (2015) (using books as an example of IP whose production costs have undergone substantial reductions).

^35^AI is already creating musical compositions—while they are drawing notice, it is not yet the case that they are overtaking humans in sales (Grow, 2021).

^36^Desai (2014).

^37^Lemley and McKenna (2020) (noting that “[i]ncumbents don't like innovation disrupting their industries,” and arguing that courts have been too receptive to incumbents' attempts to use IP law to prevent disruption because courts have failed to consider the fit between IP law's goals).

^38^Chang (2022).

^39^Office of the New York State Comptroller (2021).

^40^*Id.*, p. 9.

^41^*Id.*, p. 2.

^42^*Id.*, p. 12.

^43^McChesney (1997).

^44^The epitome of this may be Washington, DC, which pre-COVID hosted more jobs than *total* residents, including children, senior citizens and the disabled. Clabaugh (2016) (reporting Bureau of Labor Statistics finding that DC had 100,000 more jobs than residents).

^45^*See* Comptroller, p. 12.

^46^For example, where a state statute requires geographic presence in its grant of taxing authority to a city over non-resident commuters, without the commute, that taxing authority may disappear. Report, The Pew Charitable Trusts (2022).

^47^Maine Center for Economic Policy (2014).

^48^*Id*.

^49^Massachusetts Department of Revenue (2020).

^50^Shira Shoenberg (2021) (observing that “[a]lthough state clash ends, remote work issue remains”).

^51^*Id*.

^52^The White House (2022).

^53^Florida (2019).

^54^*See* Beveridge (2018) (asking the reader to “consider gasoline,” and pointing out that “[t]he demand for gas is inelastic” “because we have very little ability to buy less when the price increases”).

^55^*Id*.

^56^*See* Shill, *supra* n. 16. As an example linking direct subsidization of commuting to the traditional office, *see, e.g.*, Ohio Roadwork Development Account (described as a program under the Ohio Department of Development that “induce[s] companies to move forward with capital investment and job creation” by providing money for “off-site public road improvements” connected to the company's work site).

^57^*See* Bernstein (1994) (limiting “scope to the workplace, although sexual harassment exists in schools, housing, family and quasi-family relationships, prisons, and almost any setting where people with unequal power coexist,” and observing that “[m]any countries' laws do not even acknowledge the existence of sexual harassment outside the workplace”).

^58^Balkin (1999) (arguing that “'[t]he workplace' is not a place; it is a set of social relations of power an privilege, which may or may not have a distinct geographical nexus”).

^59^*See, e.g., EEOC* v. *Mid-Am. Specialties*, 774 F. Supp. 2d. 892 (W.D. Tenn. 2011) (sexual harassment case in traditional office setup involving physical assult); *Weeks* v. *Baker* and *McKenzie*, 63 Cal. App. 4twentieth century 1128 (Cal. Ct. App. 1998) (same in traditional law office setup); *EEOC* v. *Jackson National Life Ins. Co.*, Civil Action No. 16-cv-02472-PAB-SKC (consent decree entered Jan. 7, 2020 in racial discrimination case involving physical assault in traditional office setup).

^60^Porter (2021) (arguing that COVID-19 exacerbated gender inequality in the workplace); Cahn and McClain (2020) (providing statistics on gender imbalance of effects caused by COVID-19, work-from-home and remote schooling).

^61^*Newsradio* (television series), NBC, Season 2, Episode 5 “The Shrink” (1994).

^62^*See* Mehra, *supra* n. 30 at 14–17 and 35–37 (discussing “anti-disruption,” including by government).

^63^*Id*.

^64^*Chamber of Commerce* v. *Seattle*, 890 F.3d 769 (9th Cir. 2020) (reversing District Court's dismissal of preemption claims) (Berk, 2020).

^65^Bender (2016).

^66^*N.C. Dental* v. *FTC*, 574 U.S. 494 (2015); *Phoebe Putney Health Sys., Inc*. v. *FTC*, 568 U.S. 216 (2012).
